# The Effects of Resisted Post-Activation Sprint Performance Enhancement in Elite Female Sprinters

**DOI:** 10.3389/fphys.2021.651659

**Published:** 2021-03-05

**Authors:** Aleksander Matusiński, Przemysław Pietraszewski, Michał Krzysztofik, Artur Gołaś

**Affiliations:** ^1^Department of Exercise and Sport Performance, The Jerzy Kukuczka Academy of Physical Education in Katowice, Katowice, Poland; ^2^Institute of Sport Sciences, The Jerzy Kukuczka Academy of Physical Education in Katowice, Katowice, Poland

**Keywords:** sprinting, post-activation performance enhancement, post-activation potentiation (PAP), sprint training, training and development, resisted sprint training, resisted sprint

## Abstract

Considering the effectiveness of resisted sprint training, and the acute enhancement of sprinting performance through locomotor post-activation performance enhancement, the main objective of the research was to determine the acute effects of resisted activation with loads of 5, 10, and 15% body mass on sprint and flying start sprint performance in elite female sprinters using resisted drag technology system. Ten elite female sprinters (age: 23.2 ± 5.4 years, body mass: 54.2 ± 6.1 kg, height: 167.4 ± 7.3 cm, personal best for 100 m: 12.05 ± 0.56 s, and for 400 m: 53.17 ± 2.76 s) performed two unresisted 20-m sprints (from a crouched and flying start) before and after a single resisted sprint loaded with 5, 10, or 15% body mass to verify the effectiveness of the activation stimulus. Compared with pre-activation, Friedman tests showed that peak velocity increased by 1.6 ± 2.2% [effect size (ES) = 0.66], 2.3 ± 1.5% (ES = 1.33), and 0.2 ± 1% (ES = 0.09), as well as peak force by 2.8 ± 2.1% (ES = 0.49), 3.5 ± 2.3% (ES = 1), and 0.5 ± 2.4% (ES = 0.14), concomitant with a significant decreased in sprint time by −0.5 ± 1.2% (ES = −0.07), −2.5 ± 1.3% (ES = −0.64), and −1 ± 1.4% (ES = −0.36) for the 5, 10, and 15% body mass post-activation, respectively (*p* < 0.001; for all). Furthermore, the ANOVA showed that peak power increased by 2.9 ± 2.3% (ES = 0.61), 3.8 ± 2.2% (ES = 1.05), and 2 ± 7.1% (ES = 0.22) for the 5, 10, and 15% body mass resisted-conditioning activity, respectively, with no difference between the three conditions (*p* = 0.003 main effect time, no interaction). Moreover, compared with the 5 and 15% body mass trials [−1.5 ± 2% (ES = −0.44), −0.8 ± 0.8% (ES = −0.44), respectively], the ANOVA showed that flying start sprint time significantly decreased by −4.3 ± 1.1% (ES = −1.25) (*p* < 0.001, interaction effect) after a 10% body mass resisted-conditioning activity. The results of this study indicated that resisted sprints acutely enhance sprint performance; however, their effectiveness depends on the applied load. A single resisted sprint using 10% body mass is effective at inducing a potentiating effect on subsequent 20-m flying start sprint performance in elite female sprinters. Therefore, keeping in mind the optimal load, it is recommended to perform resisted sprints as a conditioning activation when seeking to acutely enhance 20-m flying start sprint performance in these athletes.

## Introduction

Sprinting as a key motor ability in numerous sport disciplines has been studied extensively from many different perspectives. Sprinters are often included in scientific investigations, which most often include acute studies of exercise metabolism (Ross and Leveritt, 2001; Gorostiaga et al., 2010), biomechanical analysis of high-velocity running (Cronin et al., 2008; Čoh and Mackala, 2013; Mackala and Mero, 2013), and alterations in stride length and stride frequency due to different training interventions (Bushnell and Hunter, 2007; Hanon and Gajer, 2009). A wide range of training interventions are used, and one of them is resistance exercise, which has been shown to be effective in both acute and chronic improvement of sprint performance (Seitz et al., 2014b; Bolger et al., 2015; Haugen et al., 2019). Resistance exercises directed at improving sprinting speed include locomotor activities and fixed plane resistance exercises, such as jump squats and different variations of the clean and jerk and the snatch (Harris et al., 2008; Bolger et al., 2015). The locomotor form of resistance sprint training includes towing a weighted sled, tire, or runs with a parachute, or some other devices that offer resistance (Upton, 2011). However, sled towing has been investigated the most (Petrakos et al., 2016). Resisted sprint training may involve towing a sled, which provides an overload through the friction between the sled and ground surface, or a modern advanced training device, which uses drag technology to provide fully controlled resistance during the movement, such as the 1,080 Sprint (Cross et al., 2018; Mangine et al., 2018; Gepfert et al., 2020).

Resisted sprint training can be considered from the standpoint of acute as well as chronic adaptive changes within the muscles, tendons, and the central nervous system (CNS). Chronic changes in trained athletes occur following several weeks of progressive training, while acute changes relate to a single training session, where the objective is to enhance performance by additional activation through a resistance exercise. Performing a resistance exercise causes contractile stimulation, followed by fatigue and potentiation (Seitz and Haff, 2016). The force that a muscle or groups of muscles produce after contractile activity is a result of the balance between fatigue and potentiation (Docherty and Hodgson, 2007). The desired state for a sprinter following a contractile stimulation is called post-activation potentiation (PAP), which enhances muscular performance (Hodgson et al., 2005; Gołaś et al., 2016). Several mechanisms have been proposed to explain the PAP phenomenon. They include phosphorylation of myosin regulatory light chains, increased recruitment of higher-order motor units, and changes in pennation angle (Sale, 2002; Robbins, 2005). However, it has been suggested that when a performance-related PAP approach is used instead of a mechanistic one, the concept of post-activation performance enhancement (PAPE) should be used (Blazevich and Babault, 2019; Prieske et al., 2020). With respect to that, we decided that the term PAPE would be appropriate for this study.

Resisted sprint training has a strong transfer between training and performance, which in addition creates appropriate conditions for using the PAPE effect. In training, this phenomenon is achieved by using a complex training set consisting of a loaded conditioning activity, followed by an explosive post-activation exercise with a similar movement pattern (Gołaś et al., 2016). Previous studies have shown a beneficial effect of prior conditioning activity on performance during throwing and jumping movements (Dello Iacono et al., 2020; Krzysztofik et al., 2020a,b). In relation to sprints, the use of towing as a conditioning activity has been shown to be effective in acute enhancement of sprint sessions (Winwood et al., 2016; Seitz et al., 2017; van den Tillaar and von Heimburg, 2017; Wong et al., 2017; Mangine et al., 2018). The protocols of the above-mentioned studies analyzed a very wide range of loads applied during conditioning activity, from 5 to even 150% of body mass. Winwood et al. (2016) found a non-significant improvement in 5-, 10-, and 15-m sprint times at 8 and 12 min after 75% but not after 150% of body mass sled pull (by ∼1.6, ∼1.8, and ∼0.8% decrease in sprint time). Similarly, Seitz et al. (2017) reported a significantly faster 20-m sprint time (by ∼1.8%) following sled pulls with a load of 75% but not after 125% of body mass in male rugby players. Interestingly, van den Tillaar and von Heimburg (2017) have indicated similar improvements in 20-m sprint time (by ∼2%) among experienced female handball players using resisted sprints with only an absolute load of 5 kg, which corresponded to ∼7.3% of average participant’s body mass. Nevertheless, the authors investigated the effectiveness of a single-load activation, and it is not certain whether a slightly lower or higher load will prove even more effective. Additionally, in each of these studies, the authors assessed the impact of the PAPE effect on the time of sprint performed from standing or crouching start, at a distance not exceeding 20 m. Only the study by van den Tillaar et al. (2018) investigated the acute effect of resisted sprints with 10% body mass upon 60-m sprint performance. Contrary to previous studies, the authors found an increase in the sprint times on average over 4.45%. As the resisted sprints were also performed at a distance of 60 m, it probably led to excessive fatigue. In studies that showed a positive effect of resisted sprints on the acute improvements of sprint performance, they were performed over a short distance, around 15-20 m (Winwood et al., 2016; Seitz et al., 2017; van den Tillaar and von Heimburg, 2017). Therefore, it is still uncertain whether the performance enhancement might be also noticed on longer distances or different phases of the sprint.

Despite wide evidence supporting the effectiveness of PAPE protocols for acute improvements in sports performance, there is still a need for studies in groups of female participants. The vast majority of investigations were performed on male participants (Whelan et al., 2014; Winwood et al., 2016; Seitz et al., 2017; Wong et al., 2017; Krzysztofik et al., 2020a,b, 2021), whereas little scientific data are available regarding the use of the PAPE effect in female participants (van den Tillaar and von Heimburg, 2017; Pajerska et al., 2020). Although some facts might predispose males to experience a greater PAPE effect than females, such as greater number of type II fibers or greater muscle strength (Hicks et al., 2001; Trappe et al., 2003; Seitz et al., 2014a), it was not proven in the literature. A meta-analysis conducted by Wilson et al. (2013) showed that the PAPE effect occurs independently of gender, which was also confirmed by subsequent studies (Ah Sue et al., 2016; Simpson et al., 2018; Harat et al., 2020). Nevertheless, there are definitely fewer studies on PAPE among women, so it is still warranted to assess within females as well as a comparison between genders.

Considering the effectiveness of resisted sprint training, and the acute enhancement of sprinting performance through locomotor PAPE, we decided to evaluate the optimal loading for the engine assisted drag technology system 1,080 Sprint. To provide a targeted and uniform stimulus for each participant, we decided to choose relative loads instead of absolute. Since the literature prescribes loads below 15% of body mass to not impair the sprint technique (Lockie et al., 2003; Alcaraz et al., 2008, 2009; Behrens and Simonson, 2011), and training loads for improvements in acceleration and maximum phase of sprint differ (Alcaraz et al., 2009), we decided to verify the acute effects of resisted activation with a load of 5, 10, and 15% body mass on the 20-m sprint and 20-m flying start sprint in elite female sprinters. Based on previous research (Winwood et al., 2016; Seitz et al., 2017; van den Tillaar and von Heimburg, 2017), we hypothesized that a resisted sprint with 10 and 15% body mass will improve the performance of the subsequent 20-m sprint from crouched and flying start, while a 5% body mass will have no effect.

## Materials and Methods

### Participants

Ten female sprinters (from 100 to 400 m), members of the national team, participated in the study. The research was carried out during a 10-day camp in the Olympic Training Center. Their average personal best for 100 and 400 m, age, body mass, and body height were 12.05 ± 0.56 s, 53.17 ± 2.76 s, 23.2 ± 5.4 years, 54.2 ± 6.1 kg, and 167.4 ± 7.3 cm, respectively. The participants were in the preparation phase to the season, in the initial mesocycle focused on short sprints and strength-power training. All of the participants declared that they were in the luteal phase of their menstrual cycle during the experiment. All of the athletes who participated in the study met the following criteria of inclusion: they had been national team members for at least 2 years; they had competed at national and international levels in the last year; they had a previous experience with resisted sprint training; and they were free from any medical problems. The participants were informed verbally and in writing about the procedures, and possible risks and benefits of the study, and they provided written consent before the commencement of the study. Moreover, they were asked to maintain their normal dietary and sleep habits throughout the study and not to use any supplements or stimulants for 24 h prior to the sessions. The study received the approval of the Bioethical Committee of the Academy of Physical Education in Katowice (10/2018) and performed according to the ethical standards of the Declaration of Helsinki, 2013. To calculate the sample size, statistical software (G^∗^Power, Dusseldorf, Germany) was used. Given the study two-way ANOVA (three loads and two repeated measures), a moderate overall effect size (ES) = 0.7, an alpha error <0.05, and the desired power (1 – β error) = 0.8, the total sample size resulted in nine participants.

### Procedures

During particular trials, the 1,080 Sprint engine assisted measuring system (1,080 Motion AB, Stockholm, Sweden) was used for the precise selection of loads and variables, adapted to the diagnostics of sports training and performance (Gepfert et al., 2020). The system uses changing intelligent drag technology to provide fully controlled resistance in the resisted and assisted phases of the movement. The device can record running time with an accuracy of 0.01 s and the average and peak values of such variables as force (N), power (W), and velocity (m/s). According to the data reported by the manufacturer, the system shows high repeatability and accuracy for measuring position (±0.5%), velocity (±0.5%), and force (±4.8 N) (Bergkvist et al., 2015).

**FIGURE 1:**
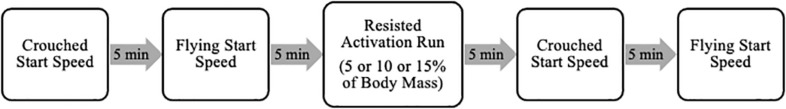
Schematic representation of the experimental sessions protocol.

The evaluations were carried out over 7 days, on Monday, Wednesday, Friday, and Sunday, with a day of rest separating the test protocols to avoid accumulation of fatigue. To prevent the influence of weather conditions (wind, temperature, etc.) on performance, the tests were performed on an indoor synthetic track. All activation, familiarization, and testing sessions were performed at the same time of the day, between 10:00 a.m. and 12:00 p.m., to avoid the influence of circadian rhythm on performance. On Monday, the participants were familiarized with the experimental loaded sprints procedure with 5, 10, and 15% body mass and were subjected to anthropometric measurements (height and weight). The participants used their track spikes during the activation protocol and during the sprint evaluations. The research protocol was always preceded by a standardized, sprint specific warm-up that was consistent with participants’ normal training habits. The test protocol for each day was identical, except for the resistance used during the activation protocol as shown in Figure 1. It consisted of two pre-activation runs from a crouched and flying start, followed by a resisted activation run with chosen load (5, 10, or 15% body mass in random order); next, the same two post-conditioning activity test trials from a crouched and flying start were performed, which were used to verify the effectiveness of the activation stimulus. Five-minute rest intervals were used between each run. This recovery interval was chosen because the participants in the current study were well-trained and the loads used during conditioning activity were likely to be low. In such situations, the evidence indicates that a 5-min post-conditioning activity recovery interval is sufficient to elicit a meaningful improvement in sprint performance (Seitz and Haff, 2016; van den Tillaar and von Heimburg, 2017). Each activation run with different resistance was performed on separate days (Wednesday, Friday, and Sunday), including a day of recovery in between. Only a single run was performed as an activation because previous studies have shown that it is sufficient to immediately improve sprint performance (Seitz et al., 2017; Mangine et al., 2018) and to focus on the effect of the load instead of volume on the results obtained. Participants started from a standing position over a distance of 20 m, and maximum acceleration throughout the entire distance is required from the participants. The participants were attached to the measuring device in the same way as during the crouched start.

**FIGURE 2:**
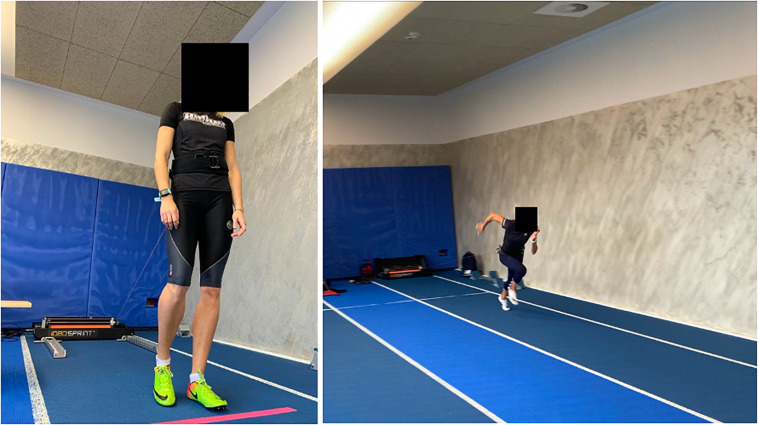
Participant Connection to the SPRINT 1080 device.

The crouched start trial evaluated sprint performance and consisted of running 20 m with maximum effort, with minimal resistance (1 kg). After receiving a verbal signal, the participants started. During the test, the participants were connected to the 1,080 Sprint measuring device with a light belt fastened around the hips, so that their movements were not restricted in any way, as shown in Figure 2. The 1,080 Sprint was firmly attached to the ground. During the 20-m sprint trial, the time (s) and the following variables were recorded in peak values: power (W), force (N), and velocity (m/s). These values were measured since the evidence suggests that sprint performance is reliant on the expression of the absolute level and balance of power, force, and velocity (Morin et al., 2011; Samozino et al., 2012). The velocity is determined through a high-resolution optical encoder measuring the position of the motor axis, which is used to calculate velocity and acceleration through differentiation of position with regard to time. The sampling frequency of position data is 333 Hz. The force is determined based on the amount of current and voltage sent to the motor by the servo drive. The actual torque of the motor shaft can then be determined, and power measures were ultimately calculated using the product of velocity and force (Bergkvist et al., 2015). However, it should be noted that the device takes into account only the force of the cord without the ground reaction force, which means that it is not the total power generated during the start of a sprint by an athlete. Based on sprint trials from the familiarization session, the intraclass correlation coefficients were 0.971 for 20-m sprint time, 0.905 for velocity, 0.931 for force, and 0.895 for power. The flying start trial was performed after a 5-min rest interval from a crouched start. This trial assessed flying start sprint time (s), a start to a time trial in which the participants are already running with maximum effort in a 20-m distance and passes the starting line, and the measurement is performed for the next 20 m. The time of the 20-m flying start sprint was recorded with a set of photocells (Witty, Microgate, Bolzano, Italy). The intraclass correlation coefficient was 0.940 for 20-m flying start sprint time.

### Statistical Analysis

All analyses were performed using SPSS (version 25.0; SPSS, Inc., Chicago, IL, United States) and were expressed as means with standard deviations (±SD). Statistical significance was set at *p* < 0.05. The Shapiro–Wilk tests were used in order to verify the normality of the sample’s data. Two-way repeated-measures ANOVA were used to compare changes in the variables pre- and post-conditioning activity under the three loaded conditions (5, 10, and 15% of body mass). ESs for main effects and interaction were estimated by calculating partial eta squared values (η^2^). Partial eta squared (η^2^) values were classified as small (0.010-0.059), moderate (0.060-0.137), and large (>0.137) (Cohen, 2013). Mauchly’s tests were conducted to test for sphericity of data; and if violated (p < 0.05), the Greenhouse–Geisser adjustment value was used. In the event of a significant main effect, *post hoc* comparisons were conducted using the Bonferroni tests. ES was determined for pairwise comparisons using Cohen,s *d*, and 95% confidence intervals were also calculated. ESs were defined as trivial (*d* < 0.20), small (*d* between 0.49 and 0.20), moderate (*d* between 0.79 and 0.50), and large (*d* > 0.80) (Cohen, 2013). However, when the normality was not confirmed, related-samples Friedman’s two-way ANOVA by ranks were used; ESs were estimated by Kendall’s coefficient of concordance. When significant, pairwise comparisons were also conducted using a Bonferroni test.

## Results

The Shapiro–Wilk tests indicated that the normality of the data was violated for sprint time, peak velocity, and peak force. Friedman’s test showed differences in pre- and post-conditioning activity values for sprint time (test = 23.96; *p* < 0.001; Kendall’s *W* = 0.479), peak velocity (test = 22.49; *p* < 0.001; Kendall’s *W* = 0.450), and peak force (test = 24.61; *p* < 0.001; Kendall’s *W* = 0.492). Pairwise comparisons demonstrated a decrease of sprint time and increase in peak velocity values after the 10% body mass resisted-conditioning activity when compared with pre-conditioning activity values (*p* = 0.006 and *p* = 0.035, respectively) (Table 1).

**TABLE 1:** Pre- and post-conditioning activity sprint results at 20 m.

Time (s)				

Load (% of body mass)	Baseline (95% CI)	PAPE (95% CI)	ES (95% CI)	Relative effects (%)
5%	3.38 ± 0.14 (3.28 to 3.48)	3.37 ± 0.15 (3.26 to 3.47)	−0.07 (−0.94 to 0.81)	−0.5 ± 1.2
10%	3.37 ± 0.14 (3.27 to 3.47)	3.29 ± 0.11* (3.21 to 3.37)	−0.64 (−1.51 to 0.29)	−2.5 ± 1.3
15%	3.36 ± 0.13 (3.27 to 3.45)	3.32 ± 0.09 (3.26 to 3.39)	−0.36 (−1.23 to 0.54)	−1 ± 1.4
**Peak Velocity (m/s)**				
5%	7.80 ± 0.16 (7.69 to 7.91)	7.92 ± 0.2 (7.78 to 8.07)	0.66 (−0.26 to 1.53)	1.6 ± 2.2
10%	7.86 ± 0.14 (7.76 to 7.96)	8.04 ± 0.13* (7.94 to 8.13)	1.33 (0.31 to 2.24)	2.3 ± 1.5
15%	7.86 ± 0.09 (7.79 to 7.92)	7.87 ± 0.12 (7.79 to 7.96)	0.09 (−0.79 to 0.97)	0.2 ± 1
**Peak force (N)**				
5%	51.88 ± 2.9 (49.8 to 53.96)	53.32 ± 2.94 (51.21 to 55.43)	0.49 (−0.42 to 1.36)	2.8 ± 2.1
10%	52.18 ± 1.3 (51.25 to 53.11)	54 ± 2.23 (52.41 to 55.59)	1 (0.03 to 1.88)	3.5 ± 2.3
15%	51.74 ± 1.38 (50.75 to 52.73)	52.01 ± 2.25 (50.4 to 53.62)	0.14 (−0.74 to 1.02)	0.5 ± 2.4
**Peak power (W)**				
5%	377.4 ± 17.42 (364.9 to 389.9)	388.4 ± 18.81* (374.9 to 401.8)	0.61 (−0.31 to 1.48)	2.9 ± 2.3
10%	386 ± 11.28 (377.9 to 394.1)	400.6 ± 16.2* (388.9 to 412.2)	1.05 (0.07 to 1.93)	3.8 ± 2.2
15%	378.3 ± 29.32 (357.3 to 399.2)	385.3 ± 34.4 (360.7 to 409.9)	0.22 (−0.67 to 1.09)	2 ± 7.1

*Indicates a significant difference between pre- and post-conditioning activity (<0.05). PAPE, post-activation performance enhancement; ES, effect size; CI, confidence interval.

The two-way repeated-measures ANOVA indicated no interaction effect for peak power (*p* = 0.625, η^2^ = 0.051). However, there was a main effect of time for peak power (*p* = 0.004, η^2^ = 0.624). Therefore, an increase of peak power values was registered after 5 and 10% when compared with pre-conditioning activity values (*p* = 0.003 and *p* < 0.001, respectively) but not after 15% body mass resisted-conditioning activity (*p* = 0.421). Moreover, the two-way repeated-measures ANOVA indicated an interaction effect for flying start sprint (*p* < 0.001, η^2^ = 0.66). The *post hoc* comparisons showed a significant decrease of flying start sprint time after all analyzed loads—5, 10, and 15% body mass resisted-conditioning activity—when compared with pre-conditioning activity values (*p* = 0.043, *p* < 0.001, and *p* = 0.012, respectively) (Table 2).

**TABLE 2:** Pre- and post-conditioning activity flying start sprint results at 20-m sprint.

Time (s)				

Load (% of body mass)	Baseline (95% CI)	PAPE (95% CI)	ES (95% CI)	Relative effects (%)
5%	2.42 ± 0.08 (2.36 to 2.48)	2.38 ± 0.1* (2.31 to 2.45)	−0.44 (−1.31 to 0.46)	−1.5 ± 2
10%	2.41 ± 0.08 (2.35 to 2.47)	2.31 ± 0.08* (2.25 to 2.36)	−1.25 (−2.15 to −0.25)	−4.3 ± 1.1
15%	2.39 ± 0.05 (2.36 to 2.42)	2.37 ± 0.04* (2.34 to 2.4)	−0.44 (−1.31 to 0.46)	−0.8 ± 0.8

*Indicates a significant difference between pre- and post-conditioning activity (<0.05). PAPE, post-activation performance enhancement; ES, effect size; CI, confidence interval.

## Discussion

The aim of the present study was to examine if 20-m sprint performance could be enhanced by the resisted 20-m sprints as a prior conditioning activity and which load produced the highest benefits. The primary finding of this study was that in comparison with the 5 and 15% of body mass trials, the 10% body mass resisted-conditioning activity most improved flying start sprint time during subsequent 20-m sprints by ∼4.3%. Moreover, an increase in peak velocity, peak force, and peak power was found, concomitant with a reduction in 20-m sprint time in comparison with pre-activation values, with no significant difference between the three conditions. The obtained data can provide meaningful knowledge for future research and training guidance for trainers and practitioners. First, athletes and coaches could be interested to include a single resisted sprint in the warm-up or prior to competition to enhance sprinting performance afterward. Second, the potentiating effect depends on the applied load, so if coaches and athletes decide to implement this training practice, based on the results obtained in this study, it is recommended to use 10% body mass for elite female sprinters.

The vast majority of studies have focused on chronic adaptations to resisted sprint training (West et al., 2013; Petrakos et al., 2016; Alcaraz et al., 2018; Gil et al., 2018), and only a few have assessed the acute effects of resisted sprints on subsequent sprint performance (Whelan et al., 2014; Winwood et al., 2016; Seitz et al., 2017; van den Tillaar and von Heimburg, 2017; Wong et al., 2017; Mangine et al., 2018; van den Tillaar et al., 2018; Thompson et al., 2021). In addition, those that were conducted analyzed the effectiveness of a single or two very high loads (75-150% of body mass) on inducing the potentiating effect (Winwood et al., 2016; Seitz et al., 2017). Moreover, they are mainly limited to the use of sleds (Whelan et al., 2014; Winwood et al., 2016; Seitz et al., 2017; van den Tillaar and von Heimburg, 2017; Wong et al., 2017; van den Tillaar et al., 2018). When using a cable resistance device, the load is the same for an entire movement. While in the case of resisted sprint training with the use of the sled, the greatest resistance occurs at the beginning of the movement due to the force needed to overcome the static friction and then slightly decreases as force is required to continue the movement. To the best of the authors’ knowledge, only two studies to date assessed the acute effects of resisted sprints using a cable resistance device (Mangine et al., 2018). The study by Mangine et al. (2018) found no improvement in the 20-m sprint time after resisted sprints with a load equal to 5% of body mass. Moreover, Thompson et al. (2021) compared the potentiation effect of resisted sprints with a load of ∼16% of body mass (∼45% body mass sled equivalent load, as the cable device, is not dependent on sprint surface coefficient of friction) with unresisted sprints on subsequent 5– and 20-m sprint performance in varsity-level sprinters. The authors found that resisted and unresisted sprints are ineffective in inducing acute sprint performance enhancement. Nevertheless, the authors examined the activation effectiveness of a single load and 3-min (Mangine et al., 2018) or 5-min (Thompson et al., 2021) rest intervals between sprints. Our study showed the greatest improvement in sprint evaluated at 20 m, after applying a load equal to 10% of body mass (∼2.5% decrease in sprint time). Similar to Mangine et al. (2018) and Thompson et al. (2021), the results of this study also showed that the load equal to 5 and 15% of body mass did not significantly enhance 20-m sprint time (an improvement of ∼0.5 and −1%, respectively). This may indicate that, in the case of cable resisted sprints, a load equal to 5 and 15% of body mass is ineffective to elicit the meaningful PAPE effect. However, it cannot be ruled out that with the length of the rest intervals used between the conditioning activity and the sprint other than those used in this study, e.g., shorter for 5% and longer for 15%, these loads might reveal different findings.

Another explanation of the results obtained may be related to the change in the muscle activation pattern between the loads applied during conditioning activity. Studies by van den Tillaar (2020) and Zabaloy et al. (2020) showed that heavier loads led to decrease calf and hamstring muscle activity, while quadriceps muscle activity increased. Thus, it is possible that a particular sprint distance or phase requires a different load during conditioning activity. For example, Wong et al. (2017) showed that sled towing with 30% of body mass led to an improvement of sprint time by ∼4.4% in the acceleration phase over the first 5 m, but not in other split times (5-10, 10-20, and 20-30 m). In turn, the results of our study showed that even greater improvements in flying start sprint were registered after activation with a load equal to 10% body mass (by ∼4.3%). Therefore, the future research should assess whether the acute improvements will also contribute to the long-term adaptations as previously indicated that heavy-loaded resisted sled training is an effective method to improve sprint performance, specifically in the early acceleration phase (e.g., 0–5 m) (Petrakos et al., 2016; Alcaraz et al., 2018), while light loads may have a greater potential to enhance maximum velocity.

It is worth noting that another novel value of this study was the group of elite female sprinters. Studies assessing the acute impact of resisted sprints on eliciting the PAPE effect in a subsequent sprint were carried out on mixed-gender (Mangine et al., 2018) or male groups (Whelan et al., 2014; Winwood et al., 2016; Seitz et al., 2017; Wong et al., 2017), with only a single study on females (van den Tillaar and von Heimburg, 2017). A study by van den Tillaar and von Heimburg (2017) indicated that resisted sprints with an absolute load of 5 kg (the effectiveness of only this load was assessed for sprints with resistance), which corresponded to ∼7.3% of average participant’s body mass, resulted in ∼2% decrease in the 20-m sprint time among experienced female handball players. Slightly greater improvement (∼2.5%) was achieved in this study using a resisted sprint of 10% of body mass. Therefore, it seems that resisted sprint with a load of approximately 10% of body mass is a suitable conditioning activity for inducing PAPE during the 20-m sprints in well-trained females. Despite the fact that previous studies have indicated that the PAPE effect is not affected by gender (Wilson et al., 2013; Ah Sue et al., 2016; Simpson et al., 2018; Harat et al., 2020), it cannot be excluded that slightly different parameters should be used when women are trained. Bearing in mind the current findings by van den Tillaar (2020) that women should sprint with approximately 10% less loads than men during resisted sprints to have equal step and joint kinematics development, it can be speculated that also a lower load to induce PAPE might be recommended for women. Nevertheless, it requires confirmation by further studies.

The present study has several limitations that should be addressed. The first limitation of the study is that the current protocol only compares the effect of different loads during conditioning activity because the control group was not included. Moreover, the assessment of performance was based only on a single running distance (20 m), as well as a single run of the resisted-conditioning activity. Therefore, the results of the presented study do not translate to other distances. Furthermore, a constant rest interval between the conditioning activity and the subsequent sprint for all participants could have affected the results. Although the results showed a decrease in flying start sprint time, following the 10% body mass resisted-conditioning activity, the mechanistic approaches cannot be directly determined and explained. Moreover, the influence of the analyzed resistances on changes in the mechanics of the run was also not assessed. It should also be noted that the speed and time in the runs before and after activation could have been affected by the load applied during these runs, the minimum necessary (1 kg) to maintain the tension of the cord and thus to perform the measurements by the device. Furthermore, the device takes into account only the force of the cord without the ground reaction force, which means that it is not the total power generated during the start of a sprint by an athlete. Future research should also directly compare the effectiveness of resisted and assisted conditioning activity with various resistance and whether the reported acute sprint enhancement will translate into chronic performance improvement. Taking into account that the training experience and gender have a significant impact on the PAPE effect, the protocol of this study should be replicated in male sprinters and with youth female athletes with lower training experience.

## Conclusion

Findings of the current study show that a resisted-conditioning activity (10% of body mass) effectively enhances 20-m flying start sprint performance among elite female sprinters. Nevertheless, we cannot be assured that this short-term improvement will contribute to chronic adaptations. However, the employment of resisted-conditioning activity may be effective in acute sprint performance enhancement (e.g., during the warm-up prior to competition) and introduce a new training stimulus for elite sprinters.

## Data Availability Statement

The raw data supporting the conclusions of this article will be made available by the authors, without undue reservation.

## Ethics Statement

The studies involving human participants were reviewed and approved by The Bioethical Committee of the Academy of Physical Education in Katowice (10/2018). The patients/participants provided their written informed consent to participate in this study.

## Author Contributions

AM and PP: conceptualization. AG and PP: data curation and formal analysis. AM, PP, and AG: investigation. MK: writing – original draft. MK: writing – review and edited. All authors contributed to the article and approved the submitted version.

## Conflict of Interest

The authors declare that the research was conducted in the absence of any commercial or financial relationships that could be construed as a potential conflict of interest.
